# Serum Homocysteine Is Associated With HDL Only in Stroke Patients With Small Vessel Occlusion

**DOI:** 10.3389/fneur.2020.565506

**Published:** 2020-12-02

**Authors:** Yerim Kim, Ju-Hun Lee, Sang-Hwa Lee, Yeo Jin Kim, Chulho Kim, Min Uk Jang, San Jung, Jae-Sung Lim, Mi Sun Oh, Kyung-Ho Yu, Byung-Chul Lee

**Affiliations:** ^1^Department of Neurology, Kangdong Sacred Heart Hospital, Hallym University College of Medicine, Seoul, South Korea; ^2^Department of Neurology, Chuncheon Sacred Heart Hospital, Hallym University College of Medicine, Chuncheon, South Korea; ^3^Department of Neurology, Dongtan Sacred Heart Hospital, Hallym University College of Medicine, Hwaseong, South Korea; ^4^Department of Neurology, Kangnam Sacred Heart Hospital, Hallym University College of Medicine, Seoul, South Korea; ^5^Hallym Neurological Institute, Department of Neurology, Hallym University Sacred Heart Hospital, Hallym University College of Medicine, Anyang-si, South Korea

**Keywords:** HDL, cholesterol, lipid metabolism, homocysteine, brain ischemia, stroke

## Abstract

**Background:** Although controversial, homocysteine (Hcy) and lipid parameters have been associated with particular stroke subtypes. However, there are limited studies concerning the relationship between Hcy and lipid levels in acute ischemic stroke (AIS). We evaluated the impact of Hcy levels on lipid profiles in terms of specific stroke subtypes.

**Methods:** A total of 2,324 patients with first-ever AIS were recruited from two hospitals in South Korea. The exclusion criteria were as follows: (a) pre-stroke modified Rankin scale (mRS) ≥ 1, (b) undetermined or other stroke etiology, and (c) absence of Hcy data. Among the 1,580 eligible patients, the Hcy level was divided into tertile groups. Logistic regression was used to assess association of Hcy levels with lipid levels by stroke subtypes.

**Results:** Significant downward trends in total cholesterol, low-density lipoprotein (LDL), and high-density lipoprotein (HDL) were only observed in patients with small vessel occlusion (SVO) as Hcy increased. In logistic regression analysis, while in patients with SVO subtype, the highest level of Hcy tertiles (OR = 1.648, 95% CI = 1.047–2.594) was associated with the lower HDL level (≤40 mg/dL), the significance disappeared in patients with LAA and CE subtypes.

**Conclusion:** Although our study does not demonstrate causal relationship, we suggest that Hcy might play a mediating role between HDL and SVO stroke development. To clarify the role of Hcy on AIS, this study will provide academic support for designing future research.

## Introduction

Homocysteine (Hcy) is a sulfur-containing, toxic intermediate of the methionine metabolic pathway (1). Total Hcy levels are known to be associated with vascular events, mortality and morbidity after stroke (2). However, the mechanism by which Hcy affects stroke outcome is still unknown.

Although still controversial, among several stroke subtypes, Hcy levels have been shown to be associated with various small vessel disease (SVD) components, including white matter hyperintensities (WMH), lacunes, and cerebral microbleeds (3, 4). Previous studies reported a possible linkage between hyperhomocysteinemia and dyslipidemia (5–7), but studies have suggested mixed conclusions. In this regard, a recent Mendelian randomization analysis published an interesting result that genetically predicted Hcy was related to SVD but was not related to large artery atherosclerosis (LAA) or cardioembolic (CE) stroke (8). Furthermore, 1-standard deviation (SD) increase in high-density lipoprotein (HDL) was associated with a significantly decreased risk of SVD (9). Considering these results, Hcy may play an important role in a particular stroke subtype. However, there are limited academic data concerning the relationship between Hcy and lipid profiles, especially in particular stroke subtypes. The current guidelines do not recommend aggressive treatment for Hcy level. However, if the role of Hcy in a particular stroke subtype changes, to control Hcy may be the treatment option for stroke prevention.

Therefore, the aim of this study was to evaluate the impact of serum Hcy levels on lipid profiles in patients with acute ischemic stroke (AIS) by different stroke subtypes.

## Methods

### Study Population

**Figure 1:**
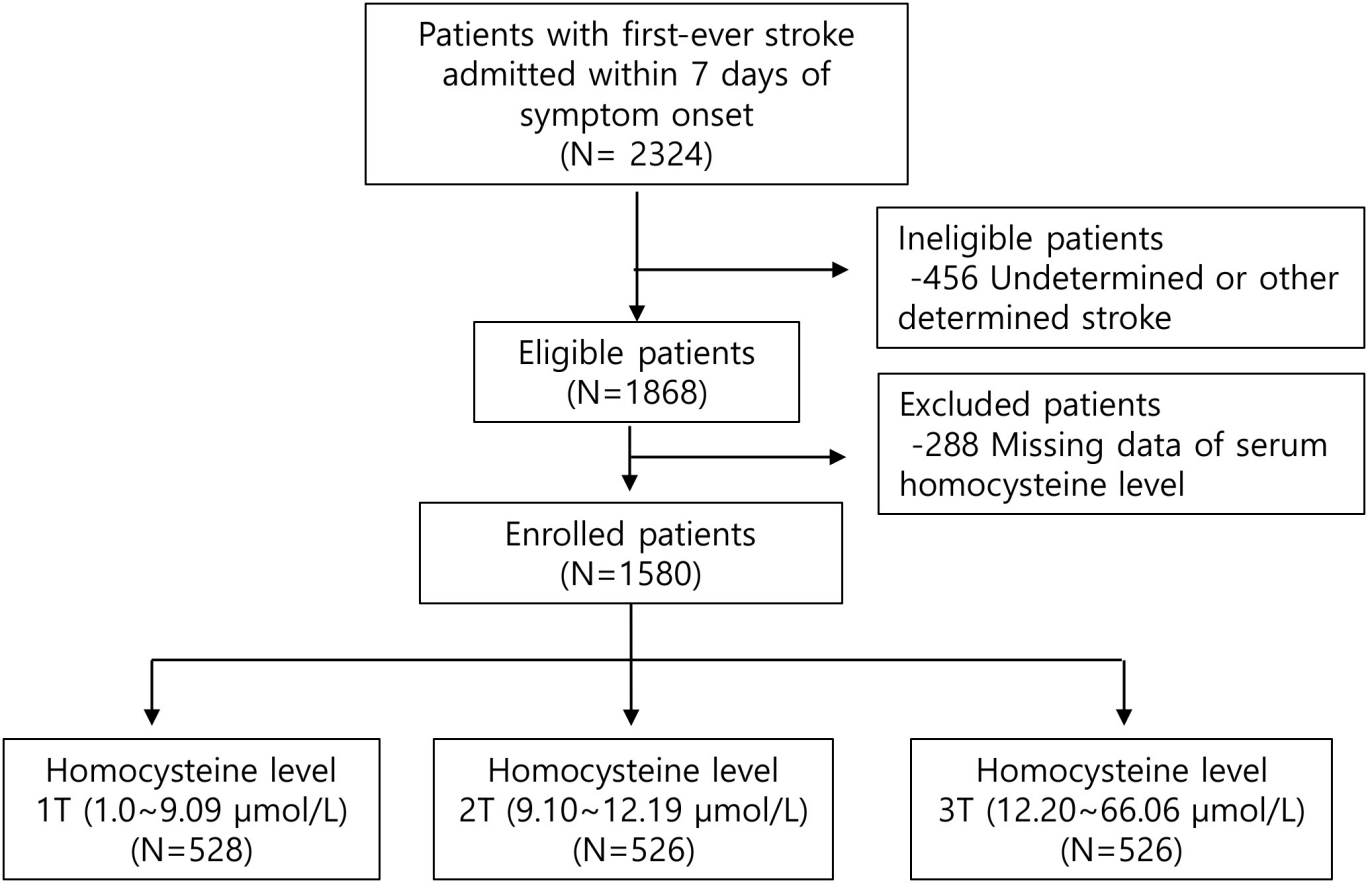
Study population flow chart.

Patients with first-ever ischemic stroke (IS) within 7 days after symptom onset who were admitted to the two tertiary university hospitals from July 2007 to January 2015 were screened. Exclusion criteria was as follows: (1) patients with previous morbidities; (2) patients with undetermined or other determined stroke etiologies; (3) subjects without acute phase Hcy levels (Figure 1).

This study was approved by the Institutional Review Board (IRB No. KANGDONG 2020-02-005).

### Stroke Subtyping

Investigators classified all stroke subtypes based on a review of original brain imaging. Trans thoracic echocardiogram was performed to evaluate the cardioembolic source in all patients. Extensive work-ups including head/neck angiography, 24-h Holter monitoring and transesophageal echocardiogram was performed. A modification of the Trial of Org 10172 (TOAST) classification was used (10). Large artery atherosclerosis was defined as symptomatic stroke with index arterial stenosis >50% or occlusion, presumably due to atherosclerosis. Cortical or cerebellar lesions and brainstem or subcortical lesions >1.5 cm in diameter on imaging are considered to be of LAA origin. Diagnostic work-ups should exclude potential sources of cardioembolism (10). According to the previous reports, CE stroke was defined as the presence of a potential moderate or high risk source of cardioembolism based on the evidence of their relative propensities for an embolus (10). To define CE etiology, we performed extensive work-ups: head/neck angiography, 24-h Holter monitoring. Transthoracic echocardiography was performed in almost all patients. Transesophageal echocardiography was administered when standard cardiac evaluation (Transthoracic echocardiography or Holter monitoring) did not reveal any CE source in high clinical suspicion of cardioembolism. Small vessel occlusion (SVO) was defined as a clinical lacunar infarction with a diameter <15 mm (11). Potential CE sources should not be present, and evaluation of the index large arteries should not demonstrate a stenosis of >50%. Other determined etiology includes subjects with rare causes of strokes, such as hematologic disorders, non-atherosclerotic stenosis, or hypercoagulable status. Undetermined etiology was defined as the cause of a stroke cannot be determined despite an extensive evaluations (negative). In others, this category includes patients with 2 or more etiologies (2 or more) or patients with incomplete evaluations (incomplete).

### Biomarkers

Serum Hcy was measured at basal level within 7 days after stroke onset using a competitive immunoassay (Immulite 2000, DPC Biermann GmbH, Bad Nauheim, Germany). Since elevated blood Hcy concentration is known to increases the risk of stroke, it is included in etiology work-ups in many tertiary hospitals. In this regard, three hospitals in this study performed this Hcy-test on all IS patients.

Levels of Hcy were divided into tertiles (1T, 1.0–9.09 μmol/L; 2T, 9.10–12.19 μmol/L; and 3T, 12.20–66.06 μmol/L). Levels of lipid profiles were measured by using enzymatic and chromatometric methods. The serum obtained was used to estimate total cholesterol, triglyceride, high-density lipoprotein (HDL), and low-density lipoprotein (LDL) by using serum separate tube.

According to the general dyslipidemia diagnosis criteria, LDL and HDL were classified into 2 groups (LDL; ≤130 vs. >130 mg/dL and HDL; >40 vs. ≤40 mg/dL).

### Stroke Scale

Initial stroke severity was assessed by the National Institutes of Health Stroke Scale (NIHSS) at admission. Functional outcome was measured at 3 months after stroke onset by using the modified Rankin Scale (mRS).

### Statistics

All statistical analyses were performed using SPSS version 21.0 (SPSS Inc., Chicago, Illinois, USA). The distribution of demographic, clinical, and laboratory data was analyzed using a χ^2^-test, Kruskal-Wallis-test, as appropriate. Since data was not normally distributed, Kruskal-Wallis-test was performed to compare whether there is a significance among groups. Values for the continuous variables are expressed as the means ± standard deviations (SDs). ^*^P for trend was calculated using linear regression. Associations between variables were assessed by using multivariable binary logistic regression analysis. Odds ratios (ORs) and 95% confidence interval (95% CI) were expressed for the results and probability values. A probability value of ≤0.05 was considered statistically significant.

## Results

Among a total of 2,324 patients without previous morbidities (previous mRS = 0), patients with undetermined or other determined stroke etiologies (*n* = 456) were excluded. Of the 1,868 eligible subjects, patients were excluded because their acute phase Hcy concentrations (*n* = 288) were not measured. Finally, a total of 1,580 patients were included in this analysis. Patients were classified as tertiles according to their Hcy level (Figure 1). Hcy levels were 1.0–9.09 μmol/L (T1), 9.1–12.19 μmol/L (T2), and 12.20–66.06 μmol/L (T3). Patients with a higher homocysteine level were older, more likely to have hypertension, a smoking habit, and higher levels of blood urea nitrogen (Table 1). Total cholesterol, LDL, and HDL levels showed a downward trend across the Hcy tertile groups. There was no difference in stroke severity or short-term functional outcome according to Hcy level.

**Table 1:** Baseline characteristics according to the homocysteine tertiles.

	**Homocysteine level 1 tertile** **(1.0–9.09 μmol/L)** **(*n* = 528)**	**Homocysteine level 2 tertile** **(9.1–12.19 μmol/L)** **(*n* = 526)**	**Homocysteine level 3 tertile** **(12.20–66.06 μmol/L)** **(*n* = 526)**	***p*-value**
Age, years (mean ± SD)	64.6 ± 13.1	65.9 ± 13.2	66.0 ± 13.3	0.001^1)^
Gender, female (*n*, %)	294 (55.7)	250 (47.5)	207 (39.4)	<0.001
TOAST, LAA (*n*, %)	202 (38.3)	208 (39.5)	227 (43.2)	0.143
SVO	200 (37.9)	198 (37.6)	164 (31.2)	
CE	126 (23.9)	120 (22.8)	135 (25.7)	
Hypertension (*n*, %)	323 (62.2)	341 (67.4)	363 (72.5)	0.002
Diabetes (*n*, %)	172 (33.8)	155 (32.4)	187 (39.5)	0.052
Dyslipidemia (*n*, %)	169 (33.7)	163 (34.2)	160 (34.8)	0.943
Smoking (*n*, %)	183 (34.7)	221 (42.0)	247 (47.1)	<0.001
Atrial fibrillation (*n*, %)	102 (20.2)	95 (19.9)	110 (24.6)	0.158
Blood urea nitrogen	16.01 ± 6.94	16.58 ± 9.24	17.54 ± 8.86	0.10^1)^
Creatinine	0.88 ± 0.67	0.90 ± 0.74	0.90 ± 0.67	0.222^1)^
White blood cell	4,653 ± 4,670	4,715 ± 4,467	4,362 ± 4,559	0.681^1)^
Hematocrit, g/dL	40.3 ± 5.3	40.7 ± 6.0	40.4 ± 6.3	0.559^1)^
Platelets	243 ± 69K	240 ± 68K	236 ± 72K	0.122^1)^
Total cholesterol, mg/dL	188.3 ± 46.4	183.7 ± 41.6	176.7 ± 45.1	<0.001^1)^
LDL, mg/dL	120.3 ± 40.5	115.4 ± 34.7	112.1 ± 35.8	0.009^1)^
LDL <= 130 mg/dL (*n*, %)	337 (64.4)	354 (67.8)	375 (72.0)	0.009^2)^
>130 mg/dL	186 (35.6)	168 (32.2)	146 (28.0)	
HDL, mg/dL	48.3 ± 13.1	46.6 ± 13.4	45.1 ± 14.4	<0.001^1)^
HDL > 40 mg/dL (*n*, %)	353 (67.6)	344 (65.8)	296 (57.0)	<0.001^2)^
<= 40 mg/dL	169 (32.4)	179 (34.2)	223 (43.0)	
Triglycerides, mg/dL	122.7 ± 76.0	131.6 ± 80.9	128.2 ± 79.0	0.080^1)^
Aspartate aminotransferase	25.0 ± 15.1	24.5 ± 12.5	24.6 ± 23.8	0.514^1)^
Alanine aminotransferase	20.8 ± 16.6	20.0 ± 14.6	19.1 ± 14.4	0.149^1)^
Fasting blood sugar	129.2 ± 59.3	118.9 ± 42.7	122.5 ± 49.9	0.066^1)^
HbA1c, %	6.51 ± 1.63	6.41 ± 1.45	6.37 ± 1.34	0.832^1)^
Prothrombin time, seconds	8.04 ± 11.89	6.57 ± 11.42	6.04 ± 12.04	**0.046**^**1)**^
Systolic BP, mmHg	147 ± 26	145 ± 25	145 ± 26	0.027^1)^
Diastolic BP, mmHg	85 ± 15	84 ± 14	84 ± 15	0.433^1)^
Admission NIHSS (IQR)	3 (1, 8)	3 (1, 5)	3 (1, 8)	<0.001^1)^
3-month mRS (IQR)	1 (0, 3)	1 (0, 3)	1 (0, 3)	0.112

*SD, standard deviation; TOAST, Trial of Org 10172 classification; LAA, large artery atherosclerosis; SVO, small vessel occlusion; CE, cardioembolic; LDL, low-density lipoprotein; HDL, high-density lipoprotein; BP, blood pressure; NIHSS, National Institutes of Health Stroke Scale; mRS, modified Rankin Scale*.

1) *Statistical significance was tested by Kruskal-Wallis-test among groups*.

2) *Linear to linear regression analysis p for trend <0.05*.

**Figure 2:**
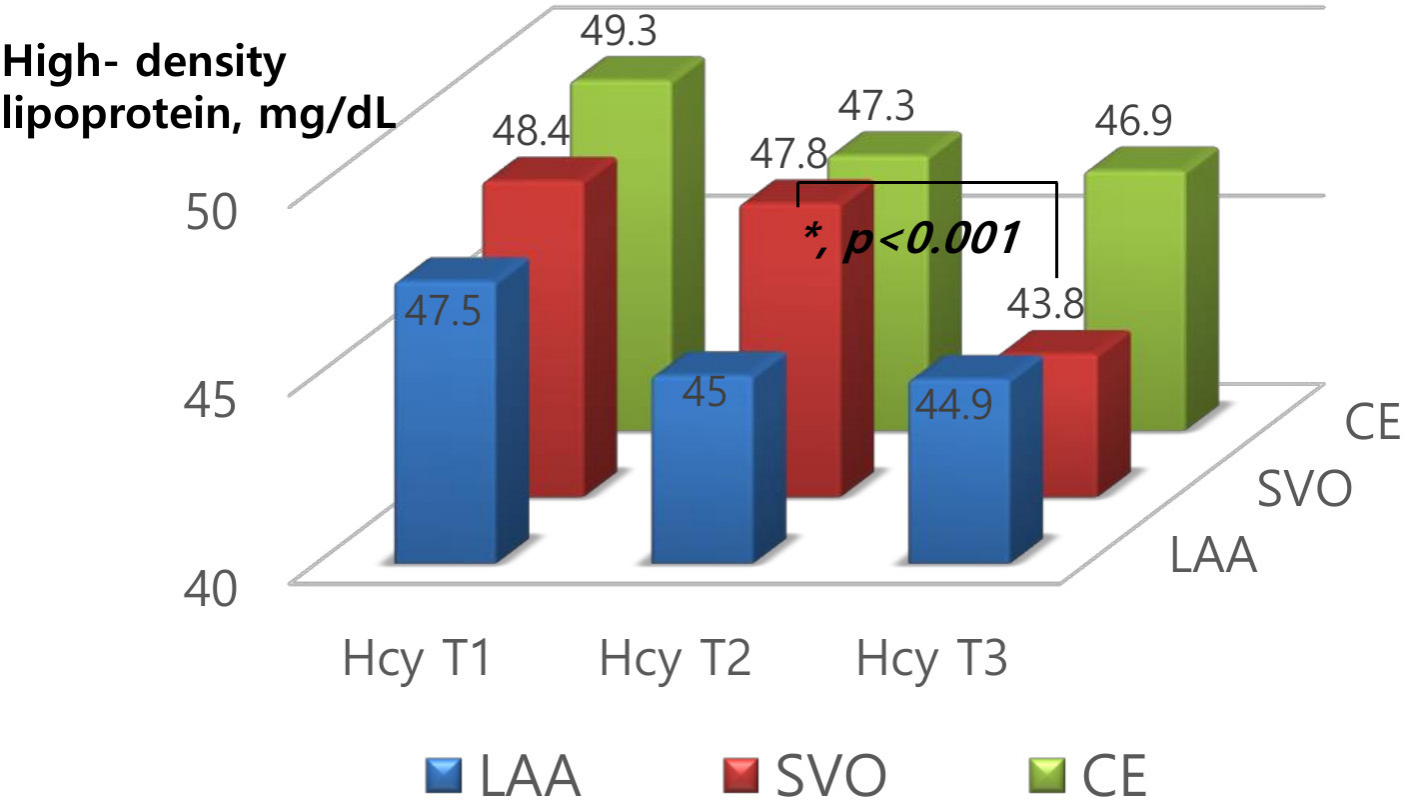
Levels of high-density lipoprotein according to homocysteine tertiles based on stroke subtypes.

According to the stroke subtypes, a downward trend of total cholesterol (*p* = 0.003), LDL (*p* = 0.021), and HDL (*p* < 0.001) was significant only in patients with SVO (Table 2 and Figure 2). A downward trend of HDL (*p* = 0.048) was significant in patients with LAA.

**Table 2:** Lipid profiles according to homocysteine level and stroke subtype.

	**Homocysteine level 1 tertile** **(1.0–9.09 μmol/L)** **(*n* = 528)**	**Homocysteine level 2 tertile** **(9.1–12.19 μmol/L)** **(*n* = 526)**	**Homocysteine level 3 tertile** **(12.20–66.06 μmol/L)** **(*n* = 526)**	***p*-value**
**TOAST, LAA**				
Total cholesterol, mg/dL	183.0 ± 44.6	183.4 ± 42.4	180.0 ± 46.7	0.420^1)^
LDL, mg/dL	118.1 ± 38.5	115.5 ± 34.1	115.2 ± 37.1	0.636^1)^
HDL, mg/dL	47.5 ± 12.7	45.0 ± 11.9	44.9 ± 13.6	0.048^1)^
Triglycerides, mg/dL	121.3 ± 69.4	136.2 ± 91.4	132.4 ± 76.8	0.174^1)^
**TOAST, SVO**				
Total cholesterol, mg/dL	203.0 ± 47.5	192.8 ± 38.7	183.6 ± 44.6	0.003^1)^
LDL, mg/dL	132.1 ± 42.4	122.0 ± 34.3	117.9 ± 34.3	0.021^1)^
HDL, mg/dL	48.4 ± 12.0	47.8 ± 14.1	43.8 ± 13.0	<0.001^1)^
Triglycerides, mg/dL	137.7 ± 85.7	141.3 ± 82.8	142.7 ± 89.1	0.825^1)^
**TOAST, CE**				
Total cholesterol, mg/dL	173.1 ± 40.7	169.4 ± 41.2	162.9 ± 39.9	0.087^1)^
LDL, mg/dL	104.9 ± 34.5	104.1 ± 33.6	99.5 ± 33.3	0.464^1)^
HDL, mg/dL	49.3 ± 15.4	47.3 ± 14.7	46.9 ± 17.1	0.297^1)^
Triglycerides, mg/dL	100.9 ± 63.6	107.6 ± 46.7	103.2 ± 62.4	0.037^1)^

*SD, standard deviation; TOAST, Trial of Org 10172 classification; LAA, large artery atherosclerosis; SVO, small vessel occlusion; CE, cardioembolic; LDL, low-density lipoprotein; HDL, high-density lipoprotein*.

1) *Statistical significance was tested by Kruskal-Wallis-test among groups*.

To clarify the relationship between Hcy and lipid profiles by stroke subtypes, we performed multivariable binary logistic regression analysis. After adjusting for age, gender, smoking, Hcy levels, prothrombin time, systolic BP, and admission NIHSS, in patients with LAA subtype, 1-elevated systolic BP (OR = 0.993, 95% CI = 0.986–1.000) and smoking (OR = 1.536, 95% CI = 1.086–2.173) was associated with HDL ≤ 40 mg/dL. In patients with SVO subtype, the highest level of Hcy tertiles (Hcy T3) (OR = 1.648, 95% CI = 1.047–2.594) and smoking (OR = 1.548, 95% CI = 1.050–2.282) was associated with the lower HDL level (HDL ≤ 40 mg/dL) (Table 3). However, the significance disappeared in patients with LAA and CE subtypes.

**Table 3:** Multivariate analysis of the associations between homocysteine levels and the lower level of high-density lipoprotein (HDL ≤ 40 mg/dL compared to HDL > 40 mg/dL).

	**Odds ratio**	***p*-value**	**95% confidence interval**
**LAA**			
Gender, female	1.331	0.121	0.927–1.912
Age	1.001	0.885	0.987–1.015
Homocysteine T1	Reference		
T2	1.116	0.610	0.733–1.698
T3	1.418	0.095	0.941–2.138
Prothrombin time	1.007	0.389	0.992–1.022
Systolic blood pressure	0.993	0.042	0.986–1.000
Smoking	1.536	0.015	1.086–2.173
Admission NIHSS	0.985	0.311	0.956–1.014
**SVO**			
Gender, female	1.123	0.551	0.767–1.646
Age	0.989	0.145	0.974–1.004
Homocystein T1	Reference		
T2	0.999	0.997	0.642–1.554
T3	1.648	0.031	1.047–2.594
Prothrombin time	0.992	0.337	0.976–1.008
Systolic blood pressure	0.998	0.602	0.992–1.005
Smoking	1.548	0.027	1.050–2.282
Admission NIHSS	0.971	0.436	0.901–1.046
**CE**			
Gender, female	0.798	0.323	0.509–1.249
Age	0.999	0.901	0.983–1.015
Homocystein T1	Reference		
T2	0.801	0.422	0.467–1.376
T3	1.151	0.605	0.676–1.961
Prothrombin time	0.996	0.720	0.977–1.016
Systolic blood pressure	0.993	0.156	0.984–1.003
Smoking	0.958	0.871	0.568–1.615
Admission NIHSS	0.994	0.713	0.964–1.025

*Adjusted for age, gender, smoking, Homocysteine levels, prothrombin time, systolic blood pressure, and admission NIHSS*.

*LAA, large artery atherosclerosis; SVO, small vessel occlusion; CE, cardioembolic; HDL, high-density lipoprotein; NIHSS, National Health Institutes of Health Stroke Scale*.

Additionally, after adjusting for multiple variables, Hcy levels did not show significant associations with LDL in all stroke subtypes (Supplementary Table).

## Discussion

Our study demonstrated that elevated Hcy levels during AIS are significantly related to decreased total cholesterol, decreased LDL and decreased HDL. According to stroke subtypes, interactions between HDL and Hcy levels were only observed in patients with SVO etiologies. To the best of our knowledge, this is the first report demonstrating relationship between Hcy and HDL considering stroke etiology.

### Associations Between Hcy and Stroke

According to previous retrospective studies, Hcy levels were significantly higher in patients with coronary artery disease and stroke (12, 13). The exact pathological mechanisms by which Hcy might contribute to stroke have not yet been elucidated. However, prothrombotic effects, pro-oxidant effects, and genetic modifications, have been suggested as hypotheses. Hcy stimulates platelet activation and affects fibrinolytic cascades (14). Vascular smooth muscle proliferation, which is directly stimulated by Hcy, is a key component of atherosclerosis (14). Furthermore, Hcy oxidizes LDL to produce thiolated-LDL, inhibits intracellular antioxidant enzymes, and reduces the bioavailability of nitric oxide. In our study, patients with higher Hcy levels had lower LDL levels. Although we cannot explain the reason, this oxidization theory may partially support the mechanism. In addition, Hcy levels were not associated with short-term functional outcome (mRS at 3 month) in this study. We think that more potent predictors such as stroke severity (NIHSS) or conventional risk factors (HTN, DM, and AF) may affect this final result.

### Associations Between Hcy and Stroke Subtypes

The relationship between hyperhomocysteinemia and stroke subtypes is inconsistent. Because IS is a heterogeneous disease, Hcy may play a specific role in different stroke subtypes. A recent study demonstrated that serum Hcy is correlated with SVO indicators, including WMH volume, cerebral microbleeds, and enlarged perivascular spaces (3). In 377 Chinese IS patients, patients with SVO etiologies with severe leukoaraiosis had the highest Hcy levels (15). In the UK black stroke population, the highest levels of Hcy were found in patients with lacunar stroke with leukoaraiosis (4). A recent meta-analysis including 18 studies with 5,088 participants found that patients with SVD subtypes have significantly elevated Hcy levels compared with the levels in controls (16). However, some studies have reported different results (4). In young and middle-aged IS patients, hyper-Hcy was more common in LAA, especially intracranial arterial stenosis, than in extracranial arterial stenosis (17). Among a total of 3,799 AIS patients, although patients with the highest Hcy levels had a 1.6-fold increased risk of mortality, this result was only significant in patients with LAA etiology (2). Despite that finding, a recent Mendelian randomization study supported that a genetically predicted 1 SD increase in Hcy was associated with SVO [odds ratio (OR): 1.34, 95% confidence interval (CI): 1.13–1.58] but was not associated with LAA or CE stroke (9). In our study, we found that elevated Hcy levels are significantly associated with lower HDL level only in patients with SVO etiology. Although we cannot explain the biologic linkage among them, we suggest that Hcy might play a key role in pathologic impact of HDL on SVO development.

### Associations Between Hcy and Lipids

It then remains to be determined why Hcy is related to HDL in patients with AIS in our study. Previous studies have shown how Hcy is associated with lipid profiles in various populations, including a community-based population (5), a coronary artery disease population (6, 18), a thyroid disease population (7) and an intensive care condition population (19). However, the exact mechanism by which Hcy affects the lipid profile in stroke patients is not well-understood. Although dyslipidemia is a well-known risk factor for stroke, the relationship between lipid profiles and IS subtypes remains controversial. In a total of 4,660 community-based Chinese people, hyperhomocysteinemia was independently related to elevated triglyceride levels and decreased HDL levels (5). In 126 patients with myocardial infarction, there was a negative correlation between HDL and Hcy and a positive correlation between LDL and Hcy (6). In 94 patients who were admitted to the intensive care unit, a negative relationship between Hcy and HDL was found with Pearson's correlation coefficient (19). However, studies examining the associations between Hcy and lipid profiles have shown mixed conclusions (20, 21). Despite that, the most consistent consensus indicates that elevated Hcy levels are associated with decreased HDL (5). This is consistent with our findings, but to the best of our knowledge, no studies have analyzed Hcy and lipid profiles in the context of stroke subtypes.

### Associations Between Lipids and Stroke Subtypes

Previous observational studies have reported discrepant conclusions concerning the relationship of lipid profiles with IS (22–24). In addition, most studies have not performed stroke subtyping. Recently, the Stroke Genetics Network found that a 1 SD genetic increase in HDL was associated with a decreased risk of SVO (OR 0.79, 95% CI 0.67–0.90) in a multivariable Mendelian randomization study (8). In this regard, although our study does not provide the causality, we suggest that Hcy might play a mediating role between HDL and SVO stroke development.

In addition to the large number of enrolled patients with MRI documentation, a strength of this study is that it is the first study to investigate which lipid parameters are associated with Hcy, considering stroke subtypes. Since stroke etiology is heterogeneous, it is very helpful to determine which biomarkers should be treated. Our findings may have novel implications that increased Hcy may be a useful biomarker linked to HDL, at least in SVO subtypes. Despite this novel strength, there are some limitations to this study. First, this is a retrospective observational study, so it does not demonstrate causal relationships between Hcy, lipids, and stroke subtypes. However, this study will provide academic support for designing future research. Second, the timing of Hcy and lipid measurements is important because plasma levels can change. Although the measurement time was not the same, the blood sample was collected the day after admission within 1 week of the AIS. Third, in patients with LAA etiology, patients with higher Hcy levels had lower LDL levels. According to prior report, Hcy can oxidize LDL and combine with LDL particles to form Hcy-thiolactone (14, 18). It may affect the ambiguous result, but a lower LDL level might be due to previous statin use. Unfortunately, we did not evaluate the medication history. Finally, the result cannot be generalized to other cohorts.

## Conclusion

In conclusion, we observed that elevated Hcy levels were significantly associated with lower HDL levels only in patients with SVO subtype. Consequently, the findings in the present study might suggest that Hcy may play a mediating role between HDL and SVO stroke development. The current guidelines do not recommend aggressive treatment for Hcy level. However, if the role of Hcy in a particular stroke subtype changes, to control Hcy may be the treatment option for stroke prevention. Future studies are needed to define the role of Hcy on lipid metabolism in stroke development.

## Data Availability Statement

Data cannot be shared because there are embargoes on datasets. Anonymized data will be shared by request from any qualified investigator.

## Ethics Statement

The studies involving human participants were reviewed and approved by IRB No. KANGDONG 2020-02-005. Written informed consent for participation was not required for this study in accordance with the national legislation and the institutional requirements.

## Author Contributions

YK: study design, clinical and image data acquisition, analysis and interpretation, and primary responsibility for writing the manuscript. B-CL and J-HL: study design, data interpretation, critical revision of the manuscript for important intellectual content, and supervision of the study. S-HL, YJK, CK, MUJ, SJ, J-SL, MSO, and K-HY: data acquisition and critical. All authors contributed to the article and approved the submitted version.

## Conflict of Interest

B-CL reports the following relationships: Boehringer-Ingelheim, Bayer, Daiichi-Sankyo, Esai, and AstraZeneca. The remaining authors declare that the research was conducted in the absence of any commercial or financial relationships that could be construed as a potential conflict of interest.
