# Supplementary material for: Serum Homocysteine Is Associated With HDL Only in Stroke Patients With Small Vessel Occlusion
Source: Front Neurol. 2020 Dec 2;11:565506. doi: 10.3389/fneur.2020.565506 (PMC7738472; doi:10.3389/fneur.2020.565506)
Supplement: Supplementary file 1 [file Table_1.DOCX]

Supplementary table. Multivariate analysis of the associations between homocysteine levels and the higher level of low-density lipoprotein (LDL >130 mg/dL compared to LDL≤130 mg/dL)

|  | **Odds ratio** | ***p*-value** | **95% confidence interval** |
| --- | --- | --- | --- |
| **LAA** |  |  |  |
| Female | 1.119 | 0.551 | 0.773-1.620 |
| Age | 0.993 | 0.347 | 0.980-1.007 |
| Homocysteine T1 | Reference |  |  |
| T2 | 0.933 | 0.749 | 0.611-1.424 |
| T3 | 0.858 | 0.471 | 0.565-1.302 |
| Prothrombin time | 0.994 | 0.467 | 0.978-1.010 |
| Systolic blood pressure | 1.001 | 0.835 | 0.994-1.008 |
| Smoking | 1.244 | 0.228 | 0.872-1.773 |
| Admission NIHSS | **1.034** | **0.023** | **1.005-1.063** |
| **SVO** |  |  |  |
| Female | 0.797 | 0.220 | 0.554-1.145 |
| Age | 0.992 | 0.281 | 0.977-1.007 |
| Homocystein T1 | Reference |  |  |
| T2 | 0.921 | 0.697 | 0.610-1.392 |
| T3 | 0.728 | 0.162 | 0.466-1.136 |
| Prothrombin time | 0.994 | 0.424 | 0.978-1.010 |
| Systolic blood pressure | 1.006 | 0.059 | 1.000-1.013 |
| Smoking | 0.753 | 0.140 | 0.516-1.098 |
| Admission NIHSS | 1.016 | 0.652 | 0.949-1.087 |
| **CE** |  |  |  |
| Female | 1.413 | 0.219 | 0.815-2.451 |
| Age | 0.986 | 0.175 | 0.967-1.006 |
| Homocystein T1 | Reference |  |  |
| T2 | 0.858 | 0.637 | 0.456-1.617 |
| T3 | 0.625 | 0.173 | 0.318-1.228 |
| Prothrombin time | 1.012 | 0.256 | 0.991-1.033 |
| Systolic blood pressure | 1.011 | 0.060 | 1.000-1.022 |
| Smoking | 0.883 | 0.700 | 0.468-1.666 |
| Admission NIHSS | 0.985 | 0.448 | 0.947-1.024 |

Adjusted for age, gender, smoking, Homocysteine levels, prothrombin time, systolic blood pressure and admission NIHSS.

Abbreviations. LAA, large artery atherosclerosis; SVO, small vessel occlusion; CE, cardioembolic; LDL, low-density lipoprotein; DBP, diastolic blood pressure; NIHSS, National Health Institutes of Health Stroke Scale
